# Using and understanding medical statistics

**DOI:** 10.1038/sj.bjc.6603786

**Published:** 2007-06-12

**Authors:** A Hackshaw

**Affiliations:** 1 University College London, London, UK

Matthews and Farewell have produced the fourth edition of their book on medical statistics. It is generally a comprehensive text that covers the main statistical methods commonly used in the medical literature. This introductory book appears to be targeted at statisticians and clinicians involved in medical research. However, it is difficult to appeal to both. While the authors have attempted to reduce the amount of algebra and technical statistical language, there is still a significant quantity of it throughout the book; so, many non-statisticians are likely to struggle with many of the sections. Calculating results by hand can be a useful tool for teaching some people, particularly those who are quite numerate. But going through the algebra for many others can often be daunting (for example, see page 151), and occasionally lead to errors if attempted by hand. With modern computers and several statistical software packages available, knowing the details of how to calculate statistical measures and test values becomes much less important for clinicians, compared with understanding which tests to use and how to interpret output correctly. Given this, I would have preferred many more examples and their interpretation, and less algebra. On the other hand, medical statisticians, particularly newly qualified ones, would find much of the book useful, as it allows them to have a greater knowledge of how various statistical measures are calculated.

The book covers many of the topics likely to be encountered in journals – data that involve counting, data that involve taking measurements on people or objects, the normal distribution, summary measures (such as means and relative risks), survival analysis, regression and correlation, analysis of variance, clinical trials, observational studies, sample size estimation and diagnostic tests.

There are some useful discussions on topics that are not commonly covered in many similar textbooks. For example, when handling multiple outcome measures in clinical trials, researchers need to be more cautious about automatically adjusting *P*-values for multiple comparisons – this is something that is often currently done but perhaps should not be, as the authors indicate (Chapter 19). Another useful summary was on the comparison of historical and randomised controls when evaluating treatments (Chapter 18). Sections like these contribute to the key strengths of the book.

Some topics could have been covered better. For example, checking whether a variable is Normally distributed (Chapter 9), which is fundamental to the application of many statistical tests, is recommended by looking at a histogram – a useful approach when there are many values in the data set. However, there are only 33 values in the diagrams in Figures 9.1a, so the histogram does not look convincingly symmetric, as concluded by the authors. From experience, a normal probability plot (where the values should lie approximately along a straight line) is much easier to examine, regardless of the number of observations, and so could have been described as well as a histogram. Another example is in Chapter 22, Diagnostic Tests. The definition of likelihood ratio (sensitivity divided by one minus specificity) is correct when the diagnostic test variable is categorical or when one is interested in the overall discriminatory power of the test. However, many screening and diagnostic tests have a continuous measurement with a normal distribution (with or without appropriate transformation), and we are often interested in estimating the risk for an individual. To do this, the other definition of the likelihood ratio is the height of the normal curve for affected individuals divided by the height of the curve for unaffected individuals, at a given value. This description could also have been presented.

Two of the new chapters that add to the value of the book are on Poisson regression (where the data are based on counts) and meta-analysis (Chapters 12 and 20). Systematic reviews (examination of several studies), which include meta-analyses, are now commonplace in medical research; so this chapter is required in the book to complement the other chapters (which tend to be associated with single studies). Because of this, Chapter 20 could have been expanded to provide examples of other outcome measures that can be combined in a meta-analysis (for example, difference between two means or risk difference), and give an example of cumulative meta-analysis.

There is no perfect book on medical statistics. The book by Matthews and Farewell should, therefore, be used to complement others that are available on the market. It is reasonably priced at about £20. Although most clinicians may not find many of the chapters easy to go through because of the algebra and technical terminology, medical statisticians and non-statisticians who want to understand the mechanics of how statistical terms are calculated are likely to find the book useful.

